# Report of the Trial of Abner Rogers, Jr., Indicted for the Murder of Charles Lincoln, Jr., Late Warden of the Massachusetts State Prison; before the Supreme Judicial Court of Massachusetts; Holden at Boston, on Tuesday, January 30th, 1844

**Published:** 1845-02

**Authors:** George Tyler Bigelow, George Bemis

**Affiliations:** Counsel for the defendant; Counsel for the defendant


*Report of the trial of Abner Rogers, Jr., indicted for the murder of Charles Lincoln, Jr., late Warden of the Massachusetts State Prison; before the Supreme Judicial Court of Massachusetts; holden at Boston, on Tuesday, January* 30, 1844. By GEORGE TYLER BIGELOW, and GEORGE BEMIS, Esqs., Counsel for the defendant.—The case of Rogers, is one of much interest to the Medical and Legal professions, from its bearing on the subject of the Medical Jurisprudence of insanity; and it is hoped that the following synopsis of the facts may prove useful, by directing attention to this important subject.

Present—Chief Justice Shaw, and Judges Wilde and Dewey.

Samuel D. Parker, Esq., the Attorney General, produced and read the conviction and sentence of Abner Rogers, Jr., as a "second comer," in the State Prison; by which it appeared that Rogers was convicted and sentenced in March, 1833, for having counterfeit money in his possession, with intent, &c., to two years hard labor in the State prison; and again, in 1838, for burglary and theft, to five years imprisonment. As a "second comer," under these two sentences, was again sentenced at the Municipal Court for the city of Boston, at the March Term, 1843, to six months additional confinement in the State prison, which last sentence he was undergoing at the time of the homicide.

On Thursday, the 15th of June, in the afternoon, Mr. Lincoln, the warden, came into the shoe-shop of the prison, where Rogers was employed, in company with a stranger.

Immediately after his entrance, and while engaged in conversation with one of the prisoners, Rogers passed across the room, took a knife from another bench, with which he approached the warden, and inflicted three wounds in quick succession; one in the small of the back, one in the back under the shoulder-blade, and another in the neck.

Without uttering a word, the warden fell into the arms of a bystander, and expired.

By the testimony of Dr. William J. Walker, physician of the prison, who made a post mortem examination of the body, it appeared that the wound in the small of the back, was slight; that the wounds under the shoulder-blade, and in the neck, were very severe; the latter, dividing the carotid artery, and injuring the windpipe.

The indictment charged the prisoner with the crime of murder, and the Attorney General stated, that in regard to the various particulars alleged, he was prepared with testimony to establish his guilt; but as the act of killing, and the manner of its accomplishment, was admitted by prisoner's counsel, the testimony relating to these points is omitted in this account.

The Attorney General farther stated to the jury, that the case depended upon their decision of the single question—

Was Abner Rogers, Jr., an accountable moral agent at the time of the homicide?

To establish this point, Dr. Walker was recalled, and stated that he had known the prisoner for a number of years, and that he saw him on the morning of the homicide, when he came to the hospital for examination.

All applicants for medical treatment come to the hospital, together, to see the physician. Rogers was among the number, on the 15th of June. He walked steadily to his seat in a quiet manner. When his turn came, he passed the door of my room in the same quiet manner toward me. I asked him what was the matter? He threw himself into gesticulations at once, put his hands up to the side of his head, and said—"I am in great distress here, I am in pain all over, and feel as if I could not govern my mind." I said to him, I understand this. If you will do your part toward meriting kind treatment, you will receive it; but if you do not, you can not receive it. He became collected immediately, was attentive to my advice, and went quietly away. After seeing me, he was sent back to the shop. My prescription, as written in the book for him, was, "Keep at work."

Between the 1st of February, 1843, and the day of the homicide, the prisoner had presented himself at the hospital as an applicant for medical treatment, twelve times; but on two occasions only, had he received any medicine. At one time liquorice was prescribed, and the other, two bread pills. I am satisfied that he was of perfectly sound mind on the 15th of June, and that he came to deceive me.

Dr. Walker further stated that he never had any conversation with the prisoner, except when he came to the hospital; that he did not feel his pulse, or examine his tongue on the morning of the fifteenth of June, and that he had not heard that he was insane, or pretended to be, prior to that time.

The defence claimed, that Rogers was insane and irresponsible at the time of the homicide ; and therefore, should be excused from the penalty of the crime of murder.

It was shown by the defence, that Rogers' general conduct had been as good as other prisoners, until within a few days of the homicide.

On Monday night, the 12th of June, he was noisy in his cell—repeted expressions of terror, as, "I shall die! I shall die!"&c. Tuesday, the 13th, appeared much agitated, and said that he was told by voices overhead, that the popo-game was to be played on him ; that checkerberry was put in his food, and that he must hold his head down and sweat it off. On Wednesday, the 14th, he repeatedly spoke of voices informing him that he was to be shut up by the warden,—of the popo-game the officers were playing on him ; and appeared very anxious about the result. At night was very noisy in his cell, for which he was showered the next morning.

Thursday, the day of the homicide, Rogers seemed agitated and restless ; did not keep at work regularly, but would stop, hold his head down, and appeared to be in a study.

An officer of the prison informed the warden that he believed Rogers' to be insane ; to which he replied, that it would not do to admit it, for fear of the effect on other prisoners.

In the afternoon he became still more excited, and repeatedly requested persons to see the warden and get him released from punishment. Although he was as often assured that no punishment was intended for him, yet it had little influence in quieting his fears, and he would immediately repeat the same request.

Between four and five o'clock in the afternoon, he went to the contractor of the shop, knelt down, and implored him to see the warden, and get him excused from punishment. At that time, his voice trembled, his hands were clasped, and he appeared in great agony. He was told to return to his work, which he did.

In a few minutes after this, Mr. Lincoln came into the room, when the fatal blows were given.

By the testimony of Abner Rogers, Sen., and other relatives, it appeared that the prisoner had fits in his infancy, that continued until he was six or seven years old; that he had been subject to turns of wakefulness at night, when he would get up and walk his room for hours in a high state of alarm. He also had one brother who was imbecile, another one who had fits; and many distant relatives, who had either been insane, or extremely nervous.

On Friday, the day after the homicide, the Rev. Louis Dwight, Secretary of the Prison Discipline Society, called to see the family of the deceased warden, and also saw the prisoner, whose appearance he thought very singular, and indicative of mental disorder. He therefore called on Dr. Bell, and induced him to visit him in the prison. The state of the prisoner's mind is fully detailed by Dr. Bell, who saw him repeatedly, and from whose testimony, as well as that of Drs. Woodward and Ray, who attended the trial, copious extracts are given. The testimony of Mr. Dwight describes the same, or similar symptoms as that of Dr. Bell, and is therefore omitted.

It may be remarked, however, that much credit is due to him, for his sagacity in perceiving Rogers' mental state, and also, for his benevolent efforts in his behalf, before, and during the trial.

Dr. Bell, testified;—I am Superintendent and Physician of the McLean Asylum, and have been such seven years and upwards. I went to see the prisoner, Rogers, at the solicitation of the Rev. Mr. Dwight, on Saturday morning, the 17th, at an early hour. I had no previous knowledge of the defendant. After some delay at the prison we were invited by the deputy-warden and chaplain to go to his cell. The deputy-warden unlocked the door, and we heard a momentary outcry, indicative of fear and affright. It seemed like that of a person just roused from sleep, and I satisfied myself that this was the case. The conversation at first was between him and Mr. Curtis, the chaplain, about sending for his father. His manner during this, was calm, composed and natural. Mr. Dwight, I think, then asked him why he killed Mr. Lincoln? Upon this subject, he began at once a strain of incoherent and wild remarks, showing plainly, that his was a case of real, or highly simulated, insanity. These he continued until I designedly turned the conversation to other subjects. His detail related to certain voices which he had heard at various times, and from several of his fellow convicts; and which announced to him, that he was to be the object of the injurious treatment or influences of the warden. The warden had learned from Mr. Curtis, as I gathered it, a knowledge of the popo-game, which Mr. Curtis had himself brought from the Auburn prison, and this was to be practiced upon *him.* The warden was also going to make another injurious attempt upon him, by putting checkerberry into his food. I asked him to explain what he meant by the popo-game. He replied, that they drove you round and round your cell; that he had stood it three nights, but that it would be impossible for him to hold out twenty-four hours longer.

I asked him what he meant by *checkerberry?* whether it was the common evergreen that grows in the woods? He said it wasn't *that,* but something that set your brain in a whirl in a moment. He said that these voices came to him, principally, from the top of his cell; some of them were in the language of reproach; as, "damn you, they'll kill you!" others were commisserative; as, "Rogers you have got to die,"—"you'll never get out of prison until you are carried out feet foremost." When the conversation was turned to other subjects, he conversed naturally in relation to them, showing no disposition to recur to these troubles. I counted his pulse twice at this interview, and found it over one hundred at each time. His tongue was slightly coated, his head cool, face shrunken, and no intolerance of light in the somewhat dark place where we were.

When he began on the popo-game and the injuries practised on him, his manner was exceedingly rapid, repeating sometimes three or four times with great volubility and rapidity, such phrases as, "tread up"—"tread up"—"damn you they'll kill you;" this, spoken with a loud voice, and great intensity. This interview continued one hour and a quarter.

I saw him again the Tuesday following, the 20th. His general manner and appearance were about the same. But there was a very considerable difference in his manner of treating these imaginary communications. His conversation was at first natural and calm. When it turned on the subject of the game, he resumed his old rapidity, but every now and then, threw in a sentence like this, "Well now ; I suppose there wasn't anything in all this," or "the devil had put these things into my imagination." His pulse at this interview was about one hundred. On turning the conversation to other points, he talked freely, and with propriety, making no illusion to the subject of the voices, which was not again referred to, at this interview.

Friday of the same week, the 23d, I saw him again, and let him know who I was, and the object of my visits ; that I came to ascertain the state of his mind. I told him this, to see if he would affect any different symptoms, or exaggerate those he had already shown. His conversation was essentially the same as before : not introducing the subject of these voices himself, and explaining them as having been merely his own imaginations, or put into his head by the devil. I tried to induce him to treat them as realities, by speaking of them as such myself ; but could not succeed. I would say to him, "When you heard Cole say" thus and so. He would reply, "I dont suppose there was any thing at all in that." He once said that he still thought his life in danger. His pulse at this time was one hundred. Dr. Bell saw the prisoner twice after this, at which times he spoke of the voices only when questioned.

At the first interview I had with him, he gave an account of trying to sweat the checkerberry out of him by hanging his head over the bed ; and of his distress in finding the blood rushing up from his heart to his head. The first three interviews were from an hour and a quarter, to an hour and a half, each. The two last less protracted.

On Saturday, he said, if he had killed Mr. Lincoln, as it was said he had, it was because this popo-game had been played upon him.

I have attended the trial and heard all the evidence that has been given in the case. As to my opinion. At the time of commencing these investigations, and until I came into court, I knew nothing of the opinions of others. I had no impression upon my mind at the time of going to the prison, but that the act was a wilful murder.

Judging from my observations alone, I have come to the conclusion, that the case is one of real, and not simulated, insanity. That the defendant was laboring under that species of insanity, which is accompanied with a belief of hearing false voices, or hallucination.

The great mass of evidence which I have since heard, supposing it to be true, corroborates my first opinion ; and I have heard but few things inconsistent with it. My reasons for my opinion are : that the form of insanity, with which this individual appeared to be afflicted, is one not generally recognized as insanity in the world at large, and would hardly be attempted by one competent to feign it, so likely would it be to fall short of its object,—that of convincing people that a person is insane who only hears false voices.

Yet the form of insanity is one well known and understood, and not unfrequently met with, by those who have the care of the insane. The delineation of it by this man, if it were feigned, was consistent, and not mingled with any symptoms which do not belong to this type of disease.

The communications reached him solely through one of the senses—that of hearing; and he never pretended that any other was affected. I have never known this form of insanity simulated.

The probabilities of a lucid interval in this case, between the time of the occurrence of the facts testified to immediately preceding the homicide, and the homicidal act, are very small. I should consider that if an outbreak had occurred under the circumstances here stated, it would evidence an uncontrollable impulse to violence. It is a well-settled fact, that after a paroxysm of violence, the insane appear comparatively calm and tranquil.

Dr. Bell here stated a case, illustrative of the last observation, and of the transitory character of the disease in some instances. A young man, who had been under his care, killed his father in a paroxysm of insanity, supposing he was the devil.

The young man for a short time previous to the act, had shown some slight symptoms of depression, or mental alienation, but nothing of an alarming charicter.

One day his father invited him to go out and work at making hay. While so engaged, as the father was stooping to go through a pair of bars, the son struck him over the head with repeated blows of a pitch-fork, and killed him.

No legal proceedings were had against him. But being brought to the hospital immediately after, (within a week,) he then appeared calm, recognized his delusion, and never showed signs of insanity afterwards.

Dr. Bell presented many other interesting illustrations and remarks, which are here omitted.

(In answer to a question from counsel for defendant,)—I consider the present, as a case of positive and decided disease.

Samuel B. Woodward, testified;—I am superintendent of the State Lunatic Hospital at Worcester, and have been so upwards of eleven years. Have been Visiting Physician and Director of the Lunatic Retreat of Hartford for ten years; and six years was Physician of the Connecticut State Prison. In the course of my experience, I have had upwards of two thousand cases of insanity, more or less under my charge, and have attended many others besides.

I have attended during this trial, and heard all, or nearly all the testimony which has been given.

Taking the facts testified to, to be true, I am of opinion that the prisoner was insane at the time of the homicide. I think his case would generally be considered one of monomania arising from hallucinations.

My reasons for this opinion are, that, in the first place, the form of the insanity is one very difficult of simulation, and but very little known in the community. Then, as shown in the prisoner's symptoms, was very coincident in all its bearings with the cases which we have; very much so. I have never known or heard of this form of insanity being simulated.

The calmness of the defendant after the act, coincides with common experience. The outbreak of an insane person seems a safety-valve by which to let off his accumulated excitement.

The outbreak or apparent commencement of the disorder, is frequently abrupt, and instantaneous. The other faculties of the mind than those affected, may remain comparatively vigorous. Cases of as short duration as the present, are not infrequent, though they can hardly be called common.

(Interesting cases illustrative of this, and other points, were mentioned by Dr. Woodward during the examination.)

It is not uncommon for the insane, or those whose delusion consists in hearing false voices, to have a passing consciousness of their delusion, and yet, immediately after, recur to the same delusion.

My view of this case is, that Rogers, seeing Mr. Lincoln enter with a stranger, and imagining that his time had come for punishment, felt an irresistible impulse to the homicidal act. My experience would lead me to think that all his thoughts were engrossed by this one idea of punishment, and that his other controlling motives, for the time being, ceased to act.

Isaac Ray, testified ;—I am Superintendent of the Maine State Hospital; have been such for about three years past. In that capacity I have had considerable experience in cases of insanity, and have otherwise devoted a good deal of attention to the study of the subject, particularly to its medical jurisprudence. I am author of the Medical Jurisprudence of Insanity, bearing my name.

I have attended during this trial, and heard all the testimony which has been given.

Taking that to be true, I believe the defendant was insane at the time of the homicide. I have not heard a single fact testified to in regard to him, during the week of the homicide, which I consider incompatible with his insanity.

In regard to the reasons for my opinion, I can say but little different from what has been already said by Drs. Bell and Woodward.—One other fact in addition to what was said by them, struck me forcibly ; that there seemed to be no constant effort on his part to convey the impression that he was insane. In regard to the physical symptoms, I should say that these showed that something was the matter with the man. The state of his pulse, his coated tongue, and shrunken features, plainly showed that he was diseased in some way.

I think I never saw, heard, or read, of a case of simulation of this kind. It would be extremely difficult to counterfeit it so as not to be detected.

*Charge of Chief Justice Shaw to the Jury.*

After some preliminary remarks on the general object of punishment by law he said :—

In order to constitute a crime, a man must have intelligence and capacity enough to have a criminal intent and purpose; and if his reason and mental powers are either so deficient that he has no will, no conscience or controlling mental powers, or if through the overwhelming violence of mental disease, his intellectual power is for the time obliterated, he is not a responsible agent, and is not punishable for criminal acts.

If it is proved to the satisfaction of the jury, that the mind of the accused was in a diseased and unsound state, the question will be, whether the disease existed to so high a degree, that for the time being, it overwhelmed the reason, conscience, and judgment, and whether the prisoner in committing the homicide, acted from an irresistible and uncontrollable impulse; if so, then the act was not the act of a voluntary agent, but the involuntary act of the body, without the concurrence of a mind directing it.

The character of the mental disease relied upon to excuse the accused in this case, is partial insanity, consisting of melancholy, accompanied by delusion. The conduct may be in many respects regular, the mind acute, and the conduct apparently governed by the rules of propriety, and at the same time there may be insane delusion by which the mind is perverted. The most common of these cases is that of monomania, when the mind broods over *one idea*, and can not be reasoned out of it. This may operate as an excuse for a criminal act in one or two modes. Either the delusion is such that the person under its influence has a real or firm belief of some fact, not true in itself, but which if it were true, would excuse his act; as where the belief is, that the party killed had an immediate design upon his life, and under that belief the insane man killed him in supposed self-defence. A common instance is when he fully believes that the act he is doing is done by the immediate command of God, and he acts under the delusive but sincere belief that what he is doing is by the command of a superior power, which supersedes all human laws, and the laws of nature. Or 2d, this state of delusion indicates to an experienced person, that the mind is in a diseased state, that the known tendency of that diseased state of the mind, is to break out into sudden paroxysms of violence, venting itself in acts of homicide, or other acts of violence toward friend or foe indiscriminately, so that although there were no previous indications of violence, yet the subsequent act, connecting itself with the previous symptoms and indications, will enable an experienced person to say, that the outbreak was of such a character, that, for the time being, it must have overborne memory and reason; that the act was the result of the disease, and not of a mind capable of choosing: in short, that it was the result of uncontrollable impulse, and not of a person acted upon by motives, and governed by the will.

The questions, then, in the present case, will be these:

1st. Was there such a delusion and hallucination?

2d. Did the accused act under a false but sincere belief that the warden had a design to shut him up, and under that pretext, destroy his life, and did he take these means to prevent it?

3d. Are the facts of such a character, taken in connection with the professional witnesses, as to induce the jury to believe that the accused had been laboring for several days under monomania, attended with delusion; and did this indicate such a diseased state of the mind that the act of killing the warden was to be considered as an outbreak or paroxysm of the disease, which, for the time being, overwhelmed or suspended reason and judgment, so that the accused was not an accountable agent?

If such was the case, the accused was entitled to an acquittal; otherwise, as the evidence proves the fact of killing beyond a doubt, without provocation, by the use of a deadly weapon, and attended with circumstances of violence, cruelty, and barbarity, he must undoubtedly be convicted of wilful murder.

In the course of the charge, and in the analysis of the evidence, the Chief Justice made some remarks upon the nature of the testimony of medical witnesses, to the following effect:

The opinions of professional men on a question of this description, are competent evidence, and in many cases are entitled to great consideration and respect. The rule of law, on which this proof of the opinion of witnesses, who know nothing of the actual facts of the case, is founded, is not peculiar to medical testimony, but is a general rule, applicable to all cases, when the question is one depending on skill and science, in any peculiar department. In general, it is the opinion of the jury which is to govern, and this is to be found upon the proof of the facts laid before them. But some questions lie beyond the scope of the observation and experience of men in general, but are quite within the observation and experience of those whose peculiar pursuits and profession have brought that class of facts frequently and habitually under their consideration. Shipmasters and seamen have peculiar means of acquiring knowledge, and experience, in whatever relates to seamenship and nautical skill.

When, therefore, a question arises in a court of justice upon that subject, and certain facts are proved by other witnesses, a shipmaster may be asked his opinion as to the character of such acts. The same is true in regard to any question of science, because persons conversant with such science have peculiar means, from a larger and more exact observation, and long experience in such department of science, of drawing correct inferences from certain facts, either observed by themselves, or testified to by other witnesses. A familiar instance of the application of this principle, occurs very often in cases of homicide, when, upon certain facts being testified to by other witnesses, medical persons are asked, whether, in their opinion, a particular wound described, would be an adequate cause, or whether such wound was, in their opinion, the actual cause of death, in the particular case. It is upon this ground, that the opinions of witnesess, who have long been conversant with insanity in its various forms, and who had the care and superintendence of insane persons, are received as competent evidence, even though they have not had opportunity to examine the particular patient, and observe the symptoms and indications of disease, at the time of its supposed existence.

When such opinions come from persons of great experience, and in whose correctness and sobriety of judgment just confidence can be had, they are of great weight, and deserve the respectful consideration of a jury.

One caution, in regard to this point, it is proper to add. The professional witnesses are not to judge of the credit of other witneses, or of the truth of the facts testified to by them. It is for the jury to decide whether such facts are satisfactorily proved. The question to be put to the professional witnesses, is this: If the symptoms and indications testified to by other witnesses are proved, and if the jury are satisfied of the truth of them, whether, in their opinion, the party was insane, and what was the nature and character of that insanity; what state of mind did they indicate; and what they could expect would be the conduct of such a person, in any supposed circumstances?

The jury retired to consider the case, but afterwards came in to ask further instructions upon the two questions.

"Must the jury be satisfied, beyond a doubt, of the insanity of the prisoner, to entitle him to an acquittal?

"And what degree of insanity will amount to a justification of the offence?

On the first point, the Chief Justice repeated his foregoing remarks upon the same head; and added, that if the preponderance of the evidence were in favor of his insanity—if its bearing and leaning, as a whole, inclined that way—they would be authorized to find him insane.

On the second point, he added nothing material to the statement of the law already made.

The jury brought in a verdict of "*Not guilty by reason of insanity.*"

The Court directed the confinement of the prisoner in the State Lunatic Hospital at Worcester, where he died a violent death some months after.

The circumstances attending his death, with other particulars relative to his health, state of mind, &c., while in the Hospital, were given by Dr. Woodward in reply to a letter of inquiry, from Rogers' counsel, Messrs. Bigelow and Bemis, of Boston.

From this letter, we extract the following particulars. When Rogers came to the Hospital, he was not quite well, had a frequent pulse, and suffered from restlessness and want of sleep. On the 5th, 6th, and 8th of February, his pulse was over one hundred per minute; he was pale, and looked anxious. On the 30th of March, and a few days after, he had another turn of sleeplessness. He complained of headache, vertigo, and loss of appetite. He looked ill, and was irritable. This turn passed off, and he appeared as well as before; worked steadily every day, attended evening prayers, and religious service on the Sabbath, and was calm and attentive at both. About the 15th of May, he was again excited, had headache, frequent pulse, furred tongue, and bad taste in his mouth, which he attributed to bad food. He told an associate that the food offered him was a corpse; he could smell it. He ate little, and did not work much the week of his death; said that he saw evil spirits in his room, and smelt corpses under his bed. His suspicion that he was to be poisoned or injured in some way, became quite strong, and was increased by these delusions, which seemed to get firm hold of his mind. He did not sleep from fear, believing that constant vigilance was his only security. During this time, he looked ill, appeared anxious and distressed. He desired to go to prayers the night he made the fatal plunge, and was not particularly uneasy until the services were nearly closed. He asked an officer near him to go out with him; that is, to his room. He told him the service was nearly ended. He then applied to his attendant, who also told him the same. He said to a patient near him, in a whisper, that the room was full of dead bodies. He appeared greatly agitated, and apprehensive. Suddenly, he sprang from the window of the room, a distance of twelve or fifteen feet; and from the injury received from the fall, died thirty-six hours after, not being conscious of anything after the leap.

While at work in the shop, some days before his death, he appeared uneasy and suspicious, and looked wild and excited. He stepped into an adjoining shop, looked about anxiously, and returned to his work without speaking. During this week he appeared much as he did the week previous to the homicide. I have no idea that Rogers intended to commit suicide. His only object was to escape from dangers, which then seemed to cluster around him. He probably acted from impulse, and as is usual in such cases, was wholly regardless of the consequences of his movements at the time. After his death, some circumstances came to our knowledge, which led us to suppose that he had thought of attempting to escape: but as far as is known, he made no such attempt.

Dr. Woodward farther states, that there was a marked coincidence in his conduct immediately before the homicide and occasionally for many years previous, and after he came to the hospital; especially the week previous to his death.

These facts and his general appearance while at the Hospital, confirm the opinion expressed on the trial, that Rogers was *insane* and *irresponsible* when he committed the homicide, and when he made the fatal rush which terminated his life.

In view of the facts presented at the trial of Rogers, together with the opinions of the medical witnesses, we are not surprised at the verdict of the jury.

Taken, also, in connection with his subsequent history, as given by Dr. Woodward, and the presumption is very strongly confirmed, that the homicide was committed under the influence of an insane impulse.

Neither is it surprising, that a man, of even Dr. Walker's ability, should have misjudged in regard to the state of Rogers' mind, when he came to the hospital, on the morning of the homicide. He had just heard of his disorderly conduct in his cell, and had witnessed, as the prisoner came in, some eccentricity of manner, which induced a suspicion against his candor. He saw him but for a moment, in company with others; and believing he came to deceive him, made no particular examination of his symptoms. He did not see, or converse with the prisoner again until after the homicide, and therefore, had no sufficient opportunity to judge of his mental condition, before that occurrence. H. A. B.

*N. Y. State Lunatic Asylum.* [*Am. Journ. of Insanity.*

